# Uncovering the evolutionary origin of plant molecular processes: comparison of *Coleochaete *(Coleochaetales) and *Spirogyra *(Zygnematales) transcriptomes

**DOI:** 10.1186/1471-2229-10-96

**Published:** 2010-05-25

**Authors:** Ruth E Timme, Charles F Delwiche

**Affiliations:** 1 Cell Biology and Molecular Genetics, University of Maryland, College Park, MD, 20742, USA; 2 Cell Biology and Molecular Genetics, Maryland Agricultural Experiment Station, University of Maryland, College Park, College Park, MD 20742, USA

## Abstract

**Background:**

The large and diverse land plant lineage is nested within a clade of fresh water green algae, the charophytes. Collection of genome-scale data for land plants and other organisms over the past decade has invigorated the field of evolutionary biology. One of the core questions in the field asks: how did a colonization event by a green algae over 450 mya lead to one of the most successful lineages on the tree of life? This question can best be answered using the comparative method, the first step of which is to gather genome-scale data across closely related lineages to land plants. Before sequencing an entire genome it is useful to first gather transcriptome data: it is less expensive, it targets the protein coding regions of the genome, and provides support for gene models for future genome sequencing. We built Expressed Sequence Tag (EST) libraries for two charophyte species, *Coleochaete orbicularis *(Coleochaetales) and *Spirogyra pratensis *(Zygnematales). We used both Sanger sequencing and next generation 454 sequencing to cover as much of the transcriptome as possible.

**Results:**

Our sequencing effort for *Spirogyra pratensis *yielded 9,984 5' Sanger reads plus 598,460 GS FLX Standard 454 sequences; *Coleochaete orbicularis *yielded 4,992 5' Sanger reads plus 673,811 GS FLX Titanium 454 sequences. After clustering *S. pratensis *yielded 12,000 unique transcripts, or unigenes, and *C. orbicularis *yielded 19,000. Both transcriptomes were very plant-like, i.e. most of the transcripts were more similar to streptophytes (land plants + charophyte green algae) than to other green algae in the sister group chlorophytes. BLAST results of several land plant genes hypothesized to be important in early land plant evolution resulted in high quality hits in both transcriptomes revealing putative orthologs ripe for follow-up studies.

**Conclusions:**

Two main conclusions were drawn from this study. One illustrates the utility of next generation sequencing for transcriptome studies: larger scale data collection at a lower cost enabled us to cover a considerable portion of the transcriptome for both species. And, two, that the charophyte green algal transcriptoms are remarkably plant-like, which gives them the unique capacity to be major players for future evolutionary genomic studies addressing origin of land plant questions.

## Background

The ancestry of all living land plants (embryophytes) can be traced back to a single colonization event from a charophyte green alga. In other words, the tremendous diversity we see in land plants today--from mosses to redwoods and orchids--all descended from a single common ancestor that colonized land 430-470 million years ago [1,2]. Uncertainty remains concerning the precise relationships between embryophytes and their algal relatives [3-8], but there is no serious doubt that the origin of land plants occurred from within the charophytes. There are six orders of charophyte green algae that, when embryophytes are included, comprise the Streptophyta *sensu lato *(s.l.) [9]: the Mesostigmatales, Chlorokybales, Klebsormidiales, Zygnematales, Coleochaetales and Charales. Both phylogenetic and fossil evidence suggest that these orders are extremely old lineages, comparable in age to the land plants [2]. Therefore, an understanding of the biology of land plants based on comparative genomics would benefit greatly if data were available from these organisms. Unfortunately, in most cases the genome size is poorly characterized, the tools of molecular genetics are not well developed, or cultures are difficult to maintain. Consequently, the acquisition of genomic data from these organisms has lagged other lineages. To move toward comprehensive genomic analysis of charophytes, we undertook EST analysis of two representative charophytes, *Spirogyra pratensis *and *Coleochaete orbicularis*.

Despite there being significant genomic resources available for the broader group of green algae, including Chlorophyta, there is only one published EST library to date that directly bears on the charophytes, that of *Mesostigma viride *[10]. *Mesostigma *is a unicellular, monotypic genus that in some analyses is placed as sister to the rest of streptophytes [6,11-14], although other studies have placed it as a sister to all other green algae [15,16]. In either case, its EST library is a valuable resource for this study. Most taxonomic and ecological diversity in the green algae resides in the Chlorophyta, a large clade sister to the streptophytes. Among the important organisms in this sister clade are the model organism *Chlamydomonas reinhardtii *and the ecologically significant *Ostreococcus tauri*. Both of these organisms have fully sequenced and published genomes [17,18].

According to Darwin's centralizing theme of descent with modification, one would predict that all land plant genes should have a homolog in the charophytes unless there was horizontal gene transfer from a non-plant organism, or unprecedented neofunctionalization. However, any one lineage of charophytes might be expected to have lost or modified some of these in the 500 million years or more of independent evolutionary history that separates each lineage from embryophytes. In this context, it is to be expected that land plant genes and their associated molecular pathways either originated in the charophytes or, if more ancient, were retained along these green algal lineages leading up to the colonization of land. Thus, it is important to sample broadly among the charophytes if the homologs of key embryophyte genes are to be identified.

In recent years PCR-based approaches have been used to fish out specific land plant genes of interest in the charophyte lineages, but advances in sequencing technologies have now made it far more efficient to gather high-throughput genomic data and work backwards, using plant gene models to annotate the putative homologous genes. Sequencing expressed sequence tags (ESTs) is an efficient first pass at gathering a large portion of the genomic coding regions. We undertook here an analysis of two distantly related charophyte taxa: *Spirogyra pratensis *Transeau (Zygnematales) and *Coleochaete orbicularis *Pringh. (Coleochaetales). Both of these lineages are essential to understanding the placement of land plants in the context of their nearest living green algal relatives. In addition, evidence of land plant molecular pathways, such as the ethylene response pathway, in the charophytes would reveal the origins of key plant molecular processes.

## Results

### EST statistics

**Figure 1:**
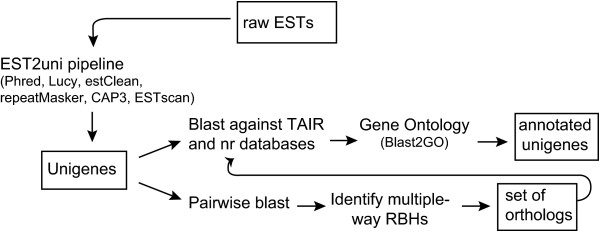
**EST analysis pipeline**. Diagram showing steps in the EST analysis pipeline. Perl-scripts were written by author unless otherwise noted. 'Raw ESTs' refer to the Sanger chromatograms plus the first clustering pass of the 454 sequences.

Our sequencing effort for *Spirogyra pratensis *yielded 9,984 5' Sanger reads plus 598,460 GS FLX Standard 454 sequences; *Coleochaete orbicularis *yielded 4,992 5' Sanger reads plus 673,811 GS FLX Titanium 454 sequences (Table 1). The average length of Sanger sequences was 915 bp (*C. orbicularis*) or 1,346 bp (*S. pratensis*) before trimming for low quality and vector sequence. The average length for the raw 454 reads differed between the older GS FLX Standard and newer GS FLX Titanium sequencing technologies of 211 and 378 bp, respectively. The 454 sequences for each species were trimmed of vector and low-quality sequences, and then clustered by Agencourt. The 454 assembly along with the raw Sanger reads were then clustered together using our in-house pipeline (Figure 1). This two-stage clustering method reduced the demand on computational power for the final assembly (CAP3 run on a desktop with 10 GB of RAM was not enough memory to cluster all the raw data together). It also allowed for careful tracking of each sequencing effort contributing to the final assembly. The average length of unigenes (contigs + singletons) in the combined 454 and Sanger sequence assembly was 813 bp for *C. orbicularis *and 571 for *S. pratensis*.

**Table 1:** EST Sequence statistics

	454 reads	5' Sanger reads	454 assembly	Sanger assembly	Combined assembly
*C. orbicularis*					
Number of reads	673,811	4992	26,373	2,455	19,313
Average length (bp)	378	915	712	721	813
GC content	47.9%	46.4%	49.2%	48.6%	49.4%
					
*S. pratensis*					
Number of reads	598,460	9984	12,357	2836	12,191
Average length (bp)	211	1346	493	845	571
GC content	42.7%	43.9%	41.1%	42.5%	41.1%

**Figure 2:**
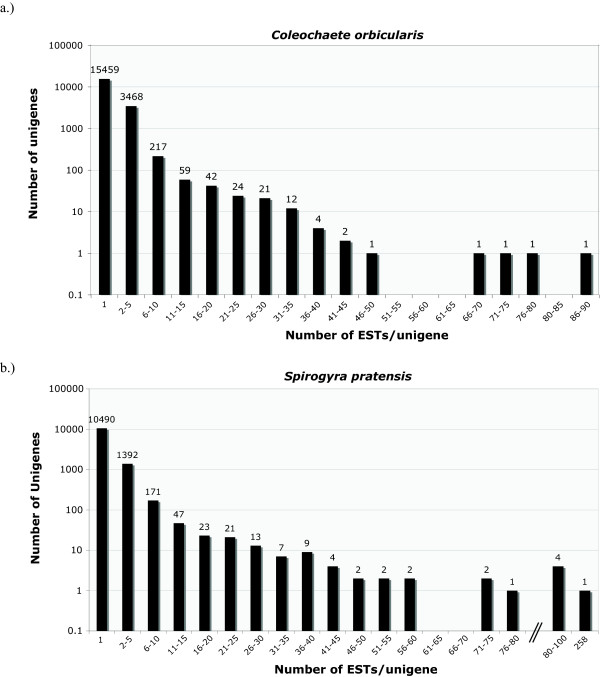
**EST Cluster Analysis**. Cluster analysis for a.) *Coleochaete orbicularis *and b.) *Spirogyra pratensis *in log scale. Each graph shows the number of ESTs per unigene. Most of the unigenes in each EST library contain a single or low number of redundant ESTs.

Summarizing the composition of the clustered ESTs is complex due to our two-step assembly procedure (the 454-only assembly followed by a combined 454 + Sanger assembly) (Table 2). Results from the *Coleochaete orbicularis *CAP3 assembly produced 19313 unigenes: 3,854 contigs and 1,549 singletons with the average length of contigs at 1,313 bp and singletons at less than half of that at 688 bp. The *Spirogyra pratensis*-combined assembly resulted in 12,191 unigenes: 1,707 contigs (average length 1,265 bp) and 10,490 singletons (average length 458 bp). The singletons category actually comprised several types of data: 454-only contigs from the first assembly, single 454 reads and single Sanger reads. Most of the singletons were in fact 454-only contigs for both EST libraries (numbers in Table 2). The number of reads per unigene was low on average, with the vast majority of unigenes comprising only one to five reads (Figure 2). A few contigs had higher numbers of reads, the most numerous being one *S. pratensis *contig with 258 reads.

**Table 2:** Contig assembly statistics. Combined 454+Sanger contig assembly statistics.

	CAP3 Contigs	Unassembled 'singletons'			
		Total	454-only contigs	454 single reads	Sanger only
*C. orbicularis*					
Number of reads	3854	15459	14919	40	500
Average length (bp)	1313	688			
					
*S. pratensis*					
Number of reads	1707	10490	9425	0	1065
Average length (bp)	1265	458			

Because ESTs are, by definition, sequences derived from messenger RNA, it follows that they should all contain at least a portion of protein-coding sequence, but several forms of contamination are possible. Our sequencing effort included 5' Sanger sequencing plus 454 sequencing, which is generated from sequencing shorter reads from sheared cloned inserts. Due to this strategy, we expected to recover the 5' (and possibly the 3') UTR and some portion of the coding sequence. However, in addition to mRNA, there is a chance of contamination in the EST library from non-coding nucleic acids, such as rRNA, tRNA and genomic DNA. These contaminants were removed from the final unigene set after ESTscan determined the putative coding regions in the sequences. By definition only the putative protein coding sequences were included in the final unigene set. Out of 12,191 total unigenes in *Spirogyra pratensis*, 25%, or 2,076, had no coding region detected. *Coleochaete orbicularis *had a lower percentage of non-coding contaminants, at 16%, or 3,080 sequences in which no protein-coding region was detected.

### Taxonomic Assignment

**Figure 3:**
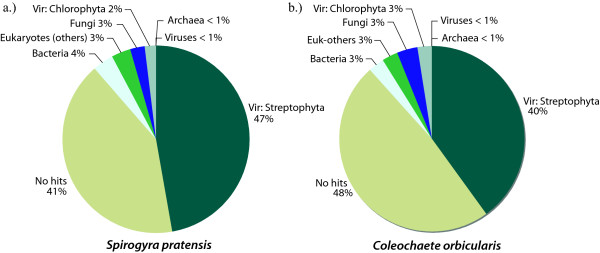
**Lineage information**. Summary of the lineage information extracted from the top BLASTx hit to NCBI's non-redundant protein database. Vir = viridiplantae. In-house perl scripts were used to query Entrez for taxonomy information.

Although every attempt was made to produce axenic cultures, including streaking and isolation off agar plates, antibiotic treatments, and zoospore isolation, we settled on "very clean" cultures. Because of this we anticipated a few contaminants based on low bacterial contamination observed when we grew the culture on agar plates. For this reason, the unigenes (all nucleotide sequences for the unique contigs and singletons) were BLASTed against the NCBI non-redundant (nr) nucleotide database using BLASTx for taxonomic lineage assignment. Eight major taxonomic categories were recovered as top hits for each *Spirogyra pratensis *and *Coleochaete orbicularis*: Archaea, Viruses, Bacteria, Fungi, Chlorophyta, Streptophyta, various other Eukaryotes, and unigenes with no significant hits (Figure 3). The proportion of hits for each species was very similar and therefore will be summarized together. About 10% of hits were possible contaminants (Bacteria, Fungi and various other Eukaryotes) and roughly 2-3% were hits to the other lineage of green algae, Chlorophyta (several chlorophyte genomes were present in GenBank at the time of this analysis). The remaining ~85% were split fairly evenly between the top hit being a streptophyte (mostly land plants) or no hits returned at all (e-value < 0.0001). A significant percentage of ESTs received no hits at all: 41% and 48% for *S. pratensis *and *C. orbicularis*, respectively. In this class of unknown unigenes, a significant portion of the sequences did not contain a protein prediction using ESTScan [19]. About half of the *Spirogyra *"no hits" and one-third in the *Coleochaete *"no hits" category were not included in the predicted protein set of unigenes, which means they are most likely a mixture of genomic and non-coding RNA contaminants. That leaves about 20% and 28% of putative novel genes in *S. pratensis *and *C. orbicularis*, respectively.

Because GenBank does not have uniform taxon sampling across the tree of life, it is important not to place too much significance on the BLAST based lineage assignment for the unigenes. For example, a small fraction of hits to metazoan taxa (included in the "various other Eukaryotes" category) most likely represents either a conserved protein domain or an ancestral gene lost in land plants, and not a metazoan contaminant. Likewise, BLAST hits to bacteria and fungi, unless evaluated individually, should be treated with caution. On the contrary, this means that up to 10% of the ESTs from each of these species could be derived from bacterial and fungal contaminants and not from the charophyte for which it's assigned. The analyses in this manuscript were largely focused on the unigenes that had similarity to land plant genomes, so it is unlikely that contaminant unigenes affected any of our major conclusions.

### Orthologous Genes

**Figure 4:**
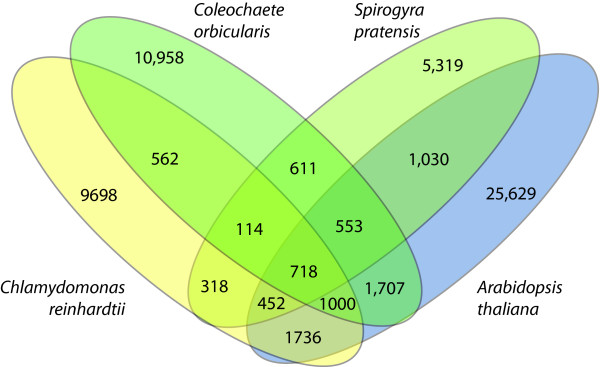
**Venn Diagram**. Venn diagram showing shared reciprocal best hits between the EST libraries and reverence genomes of *Arabidopsis *(streptophyte) and *Chlamydomonas *(chlorophyte). The numbers in each oval add up to the total number of unigenes (or genes) for each taxon.

The results of the lineage assignment introduced the question of how many genes in each EST library are actually shared with chlorophytes vs. streptophytes. To address this question, the protein predicted unigenes for *Coleochaete orbicularis *and *Spirogyra pratensis*, plus complete proteins from *Chlamydomonas reinhardtii *(a chlorophyte) and *Arabidopsis thaliana *(a streptophyte) were included in an all-vs-all pair-wise BLASTp. The reciprocal best hits (RBHs) between any two, three or four species were interpreted to be orthologs if all pair-wise possibilities in the ortholog set were each other's reciprocal best hit. All RBH overlaps are displayed in a Venn diagram (Figure 4). The total number of unigenes (or, proteins, in the case of the two reference genomes) for each species can be obtained by adding up all the numbers in the respective ovals. For example, a set of 718 unigenes are RBHs for all four species. A different set of 114 unigenes are RBHs for the charophytes + Chlamydomonas, but *not *for Arabidopsis.

Although there are many overlaps to report, the most interesting results from this analysis concerns the overlap between our species of interest and its overlap with chlorophytes *vs*. streptophytes. Looking at pair-wise comparisons only, *C. orbicularis *shares 1,707 orthologs with *Arabidopsis vs*. 562 with *Chlamydomonas*. *Spirogyra pratensis *has a similar pattern, with 1,030 shared *Arabidopsis *orthologs and 318 shared *Chlamydomonas *orthologs. This pattern holds up with three-way RBHs: *C. orbicularis *+ *S. pratensis *share 553 orthologs with *Arabidopsis *verses 114 with *Chlamydomonas*. (A list of RBHs shared by the charophytes + *Chlamydomonas *are included in Additional File 1.)

A list of genes hypothesized to be important in early land plant colonization, or "green genes," was generated from Graham et al. [20]. Each of these genes had at least one hit between the two libraries, except for the MADS domain, which had no hits in either library (Table 3). The following genes had at least one hit in both *Coleochaete orbicularis *and *Spirogyra pratensis*: RSW1 (cellulosic cell wall), GEM1/MOR1 (cytokinetic phragmoplast), CTR1 (plasmodesmata), and MERISTEM LAYER1 (multicellular sporophyte body). WUSCHEL and GNOM (asymmetric cell division) were only found in *C. orbicularis *and the expansins were only found in *S. pratensis*. A moss (*Physcomitrella patens*) and a chlorophyte (*Chlamydomonas reinhartii*) were included as references. Although all of these are significant BLAST "hits" (e-value < 1e-20) they can only be interpreted as genes of interest. Any orthology determination would need a more vigerous follow-up analysis.

**Table 3:** BLAST results for plant genes. Genes hypothesized to be important in the colonization and adaptation of land plants (Graham et al. 2000).

	Gene name	Tair Num.	***P. patens***		***C. reinhartii***		***C. orbicularis***		***S. pratensis***	
			**Hits**	**e-value**	**Hits**	**e-value**	**Hits**	**e-value**	**Hits**	**e-value**

Cellulosic cell wall	RSW1	AT4G32410	19	0.0	-	-	2	e-130	5	0.0
Cytokinetic phragmoplast	GEM1/MOR1	AT2G35630	2	0.0	1	0.0	2	e-51	1	2e-70
Plasmodesmata	CRT1	AT1G56340	6	e-62	1	e-125	5	e-141	3	3e-53
asymmetric cell division	WUSCHEL	AT1G20700	2	e-32	-	-	2	e-33	0	
asymmetric cell division	GNOM	AT1G13980	7	0.0	3	e-108	1	7e-49	0	
Specialized cells	Alpha Expansions	AT1G12560	39	e-70	-	-	0		12	3e-53
Specialized cells	Beta Expansoins	AT1G65680	4	7e-44	-	-	0		2	1e-24
Multicellular sporophyte body	MERISTEM LAYER1	AT4G21750	-	-	-	-	1	e-106	1	6e-53
Ploidy level influence in tissue differentiation	MADS domain	AT1G22130	10	e-22	-	-	0		0	

CDSs of noted genes were BLASTed against protein databases of two reference genomes, *Physcomitrella patens *and *Chlamydomonas reinhartii*, and our two charophytes, *Coleochaete orbicularis *and *Spirogyra pratensis*. Only BLASTx hits below e-20 are reported.

### Gene Ontology

**Figure 5:**
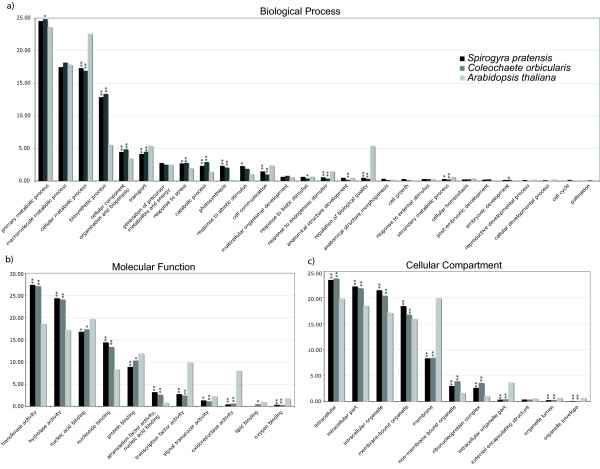
**Gene Ontology**. The relative proportion of Gene Ontologies for both charophyte ESTs shown against *Arabidopsis thaliana *proteins as a relative measure. Each graph represents 100% of the GO annotations for each species. The x-axis contains the GO categories, the y-axis shows the proportion of genes in each category. Fisher's exact test (based on a hypergeometric distribution) was used to calculate significance levels for overrepresentation or underrepresentation of each charophyte verses the reference, *A. thaliana *(* p < 0.01, ** p < 0.001).

The Gene Ontology (GO) categories recovered from Blast2GO analyses were summarized by the proportion of unigenes annotated in each GO level 3 category (Figure 5). The complete set of proteins for *Arabidopsis thaliana *was included in the analysis as a relative proportional measure since the GO assignments were all derived from *A. thaliana *annotations. Overall, *Spirogyra pratensis *and *Coleochaete orbicularis *unigene sets were remarkably similar in relative proportions, but they deviated from *A. thaliana *in several categories. Three categories stood out in biological processes (BP): both green algae were underrepresented in cellular metabolic process and regulation of biological quality (p < 0.001; p < 0.001 for *S. pratensis *and *C. orbicularis *respectively), but they were overrepresented in the biosynthetic processes category (p < 0.001; p < 0.001). For molecular function (MF), the green algae unigenes were overrepresented in transferase activity (p < 0.001; p < 0.001), hydrolase activity (p < 0.001; p < 0.001) and nucleotide binding (p < 0.001; p < 0.001), but they were underrepresented in the transcription factor activity (p < 0.001; p < 0.001) and in oxidoreductase activity categories (p < 0.00; p < 0.001). For cellular compartment (CC) annotations, the green algae unigenes were slightly over-represented in many categories, but deviated most drastically in their underrepresentation in membrane and intracellular organelle part categories (p < 0.001; p < < 0.001).

## Discussion

Both charophyte EST libraries are very land plant-like. More specifically, most of the transcriptome is more similar to embryophytes (land plants) than to other green algae in the chlorophyte lineage (the other green algal lineage sister to the streptophytes). There are some exceptions, however, which are interesting. Looking just at BLAST hits against NCBI's non-redundant (nr) protein database, 2% (214 unigenes) and 3% (488 unigenes) of the transcriptome had a top hit to a chlorophyte in *Spirogyra *and *Coleochaete*, respectively. While this is only a small fraction of total unigenes, it suggests possible shared ancestral genes between the chlorophyte and streptophyte lineages that were lost along the land plant lineage. For example, there are flagellar-associated proteins in both *Spirogyra *(two) and *Coleochaete *(13) that have top BLAST hits to *Chlamydomonas *and *Ostreococcus*, but that have no hits to *Arabidopsis *or any other land plant. Plants lost their flagellate cells in the Gymnosperms, which left Angiosperms with no motile cells. It follows that the flagellar-associated proteins would also be lost in flowering plants, infact, analysis of the much more ancestral moss genome revealed a loss of flagaller associated genes [21] that are found in other green algal and animal genomes[18]. The fact that there are no significant land plant hits suggests that these proteins were lost or underwent substantial sequence divergence in the early land plants. Also interesting is the mere presence of flagellar-associated proteins in *Spirogyra*, which lacks motile cells entirely. Containing genes for an absent structure further supports the evolutionary placement of this lineage as being derived from flagellate ancestors. An earlier hypothesis had the Zygnematales grouped with other non-flagellate organisms in a separate phylum defined by lack of motility [22], which our data clearly reject.

The overwhelming similarity to land plants also is revealed in a separate analysis in the identification of orthologous genes between our two green algae, *Chlamydomonas *and *Arabidopsis*, as shown in the Venn diagram (Fig.4). The charophytes share two to three times as many orthologs with *Arabidopsis *than they do with *Chlamydomonas*. Both the BLAST analysis and the ortholog identification reinforce the overall similarity between these lineages of green algae and land plants. Of course, a robust phylogeny of any given gene is needed before firm conclusions can be made regarding its gain or loss along the plant lineage. The point here is that even a simple BLAST analysis or ortholog identification can reveal key genes to target for follow-up study.

There are a large number of putative novel genes in both EST libraries (20% and 28% for *Spirogyra pratensis *and *Coleochaete orbicularis*, respectively). The term 'novel' is being used here to refer to genes with no obvious similarity to a model-organism sequence: i.e. new to scientists, not necessarily to the charophytes. We are classifying the unigenes as novel if they satisfy two criteria: they contain a protein-coding region predicted by ESTScan [19] and no BLAST hits returned against the NCBI non-redundant protein database. The closest fully sequenced genomes to the charophytes at the time of this publication are several embryophyte genomes, including *Physcomitrella patens *and *Arabidopsis thaliana *(which have at least 450 million years divergence with the charophytes) and *Chlamydomonas *or *Ostreococcus *in the chlorophyte lineage (which have 700-1,000 million years divergence) [2]. Because of the relative distance between charophytes and their nearest sequenced organisms, it is not surprising that they would contain genes with no obvious similarity to the current published genome-scale sequences. There are two alternative explanations for the enlarged amount of "ESTs without BLAST hits". First, the short sequences may represent non-conserved portions of genes, and, second, independent 454 sequence stretches may correspond to the same novel gene, but they do not overlap due to their limited sequence length. This last caveat would apply across the entire set of clustered set of ESTs for both species. Aside from these concerns, most of these novel sequences do appear to be unique, at least within the available data. A pair-wise BLAST between the *S. pratensis *and *C. orbicularis *sets of novel unigenes only revealed a small amount of overlap: out of 2,661 *S. pratensis *novel genes, only 24 had a hit in *C. orbicularis*. In addition, the reciprocal BLAST revealed only 27 hits in the reverse direction. None were reciprocal best hits.

The Gene Ontology analysis presents a summary of transcript diversity relative to the annotated gene diversity in *Arabidopsis*. Where there are significant differences in the proportion of genes in a category, the determination cannot be made whether there were fewer genes expressed or that there are actually fewer genes encoded in the genome--and the same goes for the relative over-expression. The observation that both charophyte green algal ESTs were so proportionally similar in their gene expression categories suggests that we are not missing any major class of gene expression and that we have captured a fairly wide range of transcript diversity representing the source genome's diversity. *Spirogyra *and *Coleochaete *are separated in evolutionary time by 500-700 million years and they do not share a similar gross morphology. But the fact that they are so alike in transcript proportion, as compared to a flowering plant, is interesting in terms of their evolutionary placement in the streptophyte lineage. The green algae showed significant difference with respect to *Arabidopsis *in most GO categories, but only a few were markedly divergent.

The under representation of charophyte genes in some of the Biological Process categories (Figure 5a) is not surprising. Although biological complexity is not easily defined, it seems clear that embryophytes have a larger number of cell types and are adapted to a wider range of environmental conditions than are charophyte algae, and consequently would be expected to have a higher proportion of genes expressed in categories involving organismal complexity. Both charophytes were significantly underrepresented in their proportion of cellular metabolic process transcripts compared to *Arabidopsis*. According to the Gene Ontology Consortium's (GOC) description of this category --the chemical reactions and pathways by which individual cells form chemical substances [23]--it stands to reason that the diversity and abundance of transcripts in this class would be higher in an organism with more diverse cell types, such as the flowering plant taxon, *Arabidopsis*. The same interpretation goes for other underrepresented categories including cell communication, response to endogenous stimulus and regulation of biological quality. The latter category had the most extreme underrepresentation of the Biological Processes categories and consequently may be one of the more interesting categories in the study of the origin of land plants. Biological quality is defined by GOC as "a measurable attribute of an organism or part of an organism, such as size, mass, shape, color, etc." The charophyte transcripts annotated to this category were similar to genes involved in cell shape, such as DIMINUTO [24], and various expansins [25]. It is logical to expect that the proportion of genes associated with cell quality would increase with organism complexity and our results are consistent with this prediction.

The explanation becomes less clear in cases where there is overrepresentation of charophyte genes in a given Biological Process category relative to *Arabidopsis*. Because we are comparing *proportions *of genes present and not the actual number found, it is possible that there might be a greater number of genes in *Arabidopsis *compared to one of the charophytes. But the proportion relative to the other categories is lower in our analysis. That said, one of the more extreme overrepresentations (over twice the proportion) of charophyte genes occurs in the biosynthetic process category, which is defined by "the chemical reactions and pathways resulting in the formation of substances; typically the energy-requiring part of metabolism in which simpler substances are transformed into more complex ones" [23]. Examples of charophyte genes that are annotated in this category are ribosomal proteins, EF-1-alpha-related GTP-binding proteins, replication proteins, translation initiation factors and ATP binding proteins. These are all core functioning genes and therefore are not expected to diversify with increasing complexity. It is likely the case that the charophyte transcriptomes contain a larger proportion of core functioning genes when compared to Arabidopsis, simply because they are lacking diversity in the other classes. Another way to look at it is that *Coleochaete *and *Spirogyra *cells are analogous to plant parenchyma and are enriched for the kinds of metabolic processes that occur in parenchyma, and not due to any trend towards increasing diversity of genes in charophyte biosynthetic processes. This probably also holds true in the photosynthesis category, where both charophytes show a significant overrepresentation of genes relative to *Arabidopsis*. An alternative hypothesis would be that, lacking structural complexity, charophytes have evolved a broader range of biochemical responses to the environment. As with most EST studies, we cannot rule out the possibility that the apparent over-representation might be a sampling artifact due to non-normalized cDNA or incomplete coverage.

Ontology by Cellular Compartment (Figure 5c) reveals one extreme difference in the membrane category whereby the charophytes are extremely underrepresented. It follows that parenchymatous tissue (i.e. the plant tissue of *Arabidopsis*) would involve an increased level of cell-to-cell communication requiring many more membrane proteins than would a filamentous organism, like *Spirogyra*, or a two-dimensional disk-shaped form, like *Coleochaete*.

Ultimately, we are interested in specific genes that allowed a lineage of charophytes to colonize land so successfully and give rise to land plants. Because the experimental approach was a survey, and because the work reported here constitutes just a part of the overall effort to gather transcriptome data for all of the charophyte orders, we are highlighting genes of interest to follow up for further study. It is encouraging that most of the genes identified by Graham et al. [20] as likely to have been important in the colonization of the land have solid hits and perhaps even true orthologs in both *Coleochaete orbicularis *and *Spirogyra pratensis *(Table 3). A couple of the more interesting genes associated with asymmetric cell division (WUSCHEL and GNOM) have hits in *Coleochaete*, but not *Spirogyra*. This is consistent with the morphology of the organisms; *Spirogyra*, an unbranched, filamentous alga does not have asymmetric cell division, but *Coleochaete *definitely does. No significant BLAST hits of the Graham et al. genes were found in the ESTs of the earliest diverging streptophyte, *Mesostigma virde*. Another interesting finding is the presence of ethylene biosynthesis and signalling pathway genes (Table 4), long thought to be unique to land plants. This finding suggests that ethylene might be utilized in the charophytes. Wang et al. [26] showed that *Chara *spp. (also a charophyte) showed ethylene binding activity similar to that of land plants, so it is not too surprising that these genes should be present in other charophyte lineages. However, to the best of our knowledge, evidence of ethylene-signalling genes in charophytes has not been shown before this study.

**Table 4:** BLAST results for ethylene pathway genes. Genes involved in ethylene biosynthesis and the ethylene response pathway.

***Gene function***	***Gene name***	***Tair Num***.	***C. orbicularis***		***S. pratensis***	
			**BLAST hit**	**e-value**	**BLAST hit**	**e-value**
			
Ethylene biosynthesis	ACO1	AT2G19590.1	1	4e-30	0	
Ethylene biosynthesis	ACS5	AT1G62960.1	1	2e-33	1	3e-32
Ethylene binding	ETR1	AT1G66340.1	0		5	e-101 - 2e-23
Ethylene binding	ERS1	AT2G40940.1	0		3	2e-61 - 6e-41
Ethylene binding	ETR2	AT3G23150.1	0		2	2e-36 - 8e-29
Ethylene binding	EIN4	AT3G04580.1	0		5	8e-51 - 3e-21
Ethylene pathway	CTR1	AT5G03730.2	5	2e-73 - 1e-43	5	9e-80 - 1e-42
Ethylene pathway	EIN2	AT5G03280.1	1	1e-18*	0	
Transcription factor	EIN3	AT3G20770.1	1	2e-73	1	e-128
Transcription factor	ERF1	AT5G47880.1	2	0.0 - 4e-43	1	e-147
Ethylene pathway	EBF1	AT2G25490.1	1	9e-73	1	3e-94
Ethylene pathway	RTE1	AT2G26070.1	0		0	

* indicates the ethylene binding domain (EBD) is present in the BLAST hit.

## Conclusions

We analyzed EST data from two species of charophyte green alga, *Spirogyra pratensis *and *Coleochaete orbicularis *as a first step toward complete genome analyses. Algal cultures were grown and collected in various life stages as well as at different times during the day. All of these factors were part of an effort to maximize the total number of transcripts available for each pulled EST library. We combined both Sanger and 454 sequencing technologies to obtain as much sequence coverage as possible. Both of the resulting EST libraries gave a nice diversity of transcripts (unigenes), with the 454 sequences contributing to most of that transcript diversity. This is especially pronounced when looking at the sequencing source of the singletons: Sanger reads only contributed a small fraction of the total singletons in both EST libraries (Table 2), and the 454 data alone covered most of the sequences obtained by Sanger sequencing. Although there have been just a few 454 transcriptome sequencing efforts to date [27-31], our results suggest that for studies of this type, 454 sequencing alone could largely replace traditional Sanger Sequencing. In summary, the specific genes we mention in this paper (Tables 3 and 4), along with the Gene Ontology analysis and ortholog identification, reinforce our finding of the overall genomic similarity that these two charophytes have to land plants.

## Methods

### Algal sampling and material collection

The following criteria were used for our taxon sampling: the availability of cultures and ease of cultivation, the presence of a sizable research community, the history of research with this organism, the perceived potential for development of advanced molecular tools, and their key evolutionary positions in two major charophyte orders. *Spirogyra pratensis *Transeau (UTEX LB 928) (Zygnematales) and *Coleochaete orbicularis *Pringsh. (UTEX LB 2651) (Coleochaetales) were grown up in Guillard's Woods Hole medium [32] at 18°C and a 12:12 LD photoperiod with a photon flux of 180-200 μmol s^-1 ^m^-2^. Cultures were harvested during log phase growth in a variety of conditions to maximize the diversity of transcripts: at intervals at 7 am, 12 pm, 4 pm and 9 pm; after sitting in a dark enclosure for 24 hours; and after being exposed to 20 min of -20°C. *Spirogyra pratensis *filaments were removed from the medium using a sterile glass hook, wicked to remove excess moisture, dropped in liquid nitrogen and stored at -80°C until RNA extraction. *Coleochaete orbicularis *cultures were scraped from the culturing flask, pelleted at 4000rpm, dropped in liquid nitrogen and stored at -80°C until RNA extraction.

### RNA isolation

Frozen tissue was ground at cryogenic temperatures using a SPEX 6770 Freezer/Mill (SPEX Certi Prep, Metuchen, NJ). The frozen ground tissue was then added to 2 × RNA extraction buffer as described in La Claire and Herrin [33], in which case a modified version of this protocol for RNA isolation was followed. After each isolation, the nucleic acid concentration and OD ratios (260/280 and 260/230) were quantified with a NanoDrop (Thermo Scientific NanoDropTM 1000 Spectrophotometer, Wilmington, DE) and the quality of RNA, or the degree of degradation, was determined by running 1 μg of total RNA on a 1.2% agarose MOPS/formaldehyde gel (Applied Biosystems/Ambion, Austin, TX) stained with ethidium bromide, then examining the rRNA banding patterns. High-quality, clean RNA was pooled until 1 mg of total RNA was reached.

### cDNA construction

Total RNA (1 mg) was shipped on dry ice to Agencourt Bioscience Corporation (Beverly, MA) where Poly(A)+RNA from total RNAs was isolated by two rounds of oligo(dT) selection with oligo(dT) coated magnetic particles (Seradyn, Inc.). From the poly(A)+RNA, cDNA libraries were constructed by using an oligo dT primer-adapter containing a Not I site and Moloney Murine Leukemia Virus Reverse Transcriptase (M-MLV RT) to prime and synthesize first strand cDNA. After the second strand was synthesized, the double stranded cDNA was size fractionated (< 1.2 kb), cloned directionally into the pExpress 1 vector and grown up in T1 phage resistant *E. coli*.

### DNA sequencing

DNA sequencing for *Spirogyra pratensis *and *Coleochaete orbicularis *included both 5 prime Sanger reads and 454 sequencing technologies: *S. pratensis *had a targeted 10,000 Sanger read plus a full plate of GS FLX Standard 454 sequences generated; *C. orbicularis *had a targeted 5,000 Sanger reads plus a full plate of GS FLX Titanium 454 sequences generated.

#### Sanger sequencing

DNA from the clones was purified using Agencourt's proprietary large-scale automated template purification systems using solid-phase reversible immobilization (SPRI). The purified DNA was then sequenced using ABI dye-terminator chemistry and then run on ABI 3730 × l (Applied Biosystems Inc, Foster City, CA) machines.

#### 454-sequencing

3-5ug of isolated DNA was nebulized to a mean size range of 3-500 bp, followed by a size selection of fragments >300 bp by column exclusion and Ampure™ (Agencourt Bioscience, Danvers, MA) isolation. The correct size selection was confirmed on an Agilent DNA 1000 LabChip. Adapters were ligated onto the fragments and selected using library capture beads. The single stranded fragments were isolated with 0.125 N NaOH, followed by neutralization with acetic acid, and purified. Single stranded library was validated qualitatively by Agilent RNA Pico 6000 LabChip and quantitatively by Invitrogen Ribogreen assay. Standard library dilutions were made according to published protocol. The library was amplified onto DNA capture beads by emulsion PCR (emPCR). DNA capture beads were collected by washes with isopropanol, Roche 454 emPCR collection reagents, and filtered syringes. Sequencing primer was annealed by thermocycler. Collected beads were quantified by counting on Beckman Multisizer. Beads for each genome were placed on the picotitre plate, sequenced on the Roche 454 GS FLX instrument, and analysed with base-calling software using default parameters.

### EST Analysis

The clustering was preformed in a two-step process. First, 454 library construction adapter sequences were trimmed, then the reads were clustered using MIRA vs 2.9.43 [34,35] with 454 EST assembly specific parameters. Second, the raw Sanger reads were combined with the 454 contigs + singleons (along with their respective quality scores) and were cleaned and clustered using the EST2uni pipeline [36]. (See Fig. 1) This pipeline used Phred [37,38], SeqClean [39], Lucy [40], and RepeatMasker [41] to remove low-quality sequence, vector contamination and low complexity regions. It then clustered the clean reads with CAP3 [42] using a 100 bp plus 95 percent identity of overlap. ESTscan [19] predicted the protein-coding regions in the contigs and singletons using the score matrix from *Arabidopsis thaliana*. At this point, the unigenes (contigs plus singletons) were available in three forms: raw nucleotides, predicted amino acids and nucleotide coding sequences (cds). Annotation of the unigenes was determined in a variety of ways. To screen for contamination, we BLASTed nucleotide unigenes against NCBI's non-redundant protein database (nr) (e-value < 0.0001), producing a lineage assignment for each EST. Taxonomic ID and lineage information for each BLAST hit was retrieved from NCBI Entrez using in-house perl scripts. Gene annotation was performed by BLASTing the predicted amino acid unigenes against the model plant, *A. thaliana *(TAIR8) (e-value < 10-6). The top hit was extracted with an in-house perl script and used for the EST unigene annotation.

The putative orthologs between our two charophytes, *Spirogyra and Coleochaete*, a land plant, *Arabidopsis thaliana*, and a chlorophyte, *Chlamydamonas reinhartii *were determined using the reciprocal best hit (RBH) criterion [43]. An all-by-all BLASTp (e-value < 1e × 10^-6^) was preformed on the proteins from each of the four taxa. In house perl scripts were used to parse the blast hits, extract the RBH pairs, and expand the pairs to include RBHs for multiple genomes/transcriptoms. The RBHs between any two, three or four species were interpreted to be orthologs if all pairwise possibilites in the ortholog set were each other's reciprocal best BLAST hit.

The BLAST results against *Arabidopsis thaliana *were also used for Gene Ontology (GO) annotation by importing the .xml BLAST results into the program Blast2GO [44]. Gene Ontology assignments were performed as follows: first, associated GO terms were mapped to the top hits of the BLAST search (against *A. thaliniana*) and, by extension, to the EST unigene; select GO annotations were chosen from the mapped group of GO terms; and finally, a combined graph analysis for Biological Processes, Molecular Function and Cellular Compartment resulted in a level three GO distribution. *Arabidopsis thaliana *GO analysis was included in the bar graph for reference and a Fisher's exact test (based on a hypergeometric distribution) was used to calculate significance levels for overrepresentation or underrepresentation of each charophyte vs. the reference, *A. thaliana*.

Arabidopsis genes of interest in early land plant evolution were obtained from http://www.arabidopsis.org/ then BLASTed against both *Coleochaete orbicularis *and *Spirogyra pratensis *(BLASTx, e-value < 1e-20). The number of BLASTx hits with e-values below this threshold were considered possible homologs for the plant gene of interest.

The individual reads comprising each EST library were deposited in GenBank. The Sanger reads are located in dbEST under the following accession numbers: *Coleochaete orbicularis *(GenBank: GW591203-GW595666), *Spirogyra pratensis *(GenBank: GW595667-GW602960). The 454 sequences are in the Sequence Read Archive (SRA): *C. orbicularis *(GenBank: SRX017046.2), *S. pratensis *(GenBank: SRX017045.2). The clustered ESTs are available for download on the author's webpage [45].

## Authors' contributions

RET cultured the algae, carried out the wet bench work, built the EST analysis pipeline and drafted the manuscript. CFD participated in the design and coordination of the study and helped to draft and edit the manuscript. Both authors read and approved the final manuscript.

## Supplementary Material

Additional file 1 **RBH ortholog sets for charophytes + *Chlamydomonas ***. Reciprocal Best Hit sets of unigenes that are shared between the Charophytes and *Chlamydomonas*, but which are absent in *Arabidopsis*. chlre: *Chlamydomonas reinhartii*; corb: *Coleochaete orbicularis*; spra: *Spirogyra pratensis*. Click here for file
